# To explore the Radix Paeoniae Rubra-Flos Carthami herb pair's potential mechanism in the treatment of ischemic stroke by network pharmacology and molecular docking technology

**DOI:** 10.1097/MD.0000000000027752

**Published:** 2021-12-10

**Authors:** Xingyu Chen, Yue Wang, Ying Ma, Ruonan Wang, Dexi Zhao

**Affiliations:** a College of Traditional Chinese Medicine, Changchun University of Chinese Medicine, Changchun, China; b Department of Encephalopathy, The Affiliated Hospital to Changchun University of Chinese Medicine, Changchun, China.

**Keywords:** enrichment analysis, Flos Carthami, ischemic stroke, molecular docking, network pharmacology, Radix Paeoniae Rubra

## Abstract

To explore the Radix Paeoniae Rubra-Flos Carthami herb pair's (RPR-FC) potential mechanism in treating ischemic stroke (IS) by network pharmacology and molecular docking technology.

The Traditional Chinese Medicine Systems Pharmacology Database was used to screen the active components of the RPR-FC, and Cytoscape 3.8 software was used to construct a network map of its active components and targets of action. The GeneCards and OMIM databases were used to identify disease targets of IS, and the common targets were chosen as research targets and imported into the STRING database to construct a protein–protein interaction network map of these targets. R language software was used to analyze the enrichment of GO terms and KEGG pathways, and explore the mechanisms of these targets. Molecular docking technology was used to verify that the RPR-FC components had a good bonding activity with their potential targets.

A total of 44 active components, which corresponded to 197 targets, were identified in the RPR-FC. There were 139 common targets between the herb pair and IS. GO functional enrichment analysis revealed 2253 biological process entries, 72 cellular components entries, and 183 molecular functions entries. KEGG pathway enrichment analysis was mainly related to the NF-kappa B signaling pathway, the TNF signaling pathway, apoptosis, the MAPK signaling pathway, the PI3K-Akt signaling pathway, the VEGF signaling pathway, etc. The molecular docking results showed the components that docked well with key targets were quercetin, luteolin, kaempferol, and baicalein.

The active components (quercetin, luteolin, kaempferol, and baicalein) of the RPR-FC and their targets act on proteins such as MAPK1, AKT1, VEGFA, and CASP3, which are closely related to IS.^1^ These targets are closely related to the NF-kappa B signaling pathway, the MAPK signaling pathway, the PI3K-Akt signaling pathway, the VEGF signaling pathway, and other signaling pathways. These pathways are involved in the recovery of nerve function, angiogenesis, and neuronal apoptosis and the regulation of inflammatory factors, which may have a therapeutic effect on IS.

## 1 Introduction

Despite new developments in treatment strategies, stroke is still one of the main causes of death and disability worldwide.^[1]^ The incidence, disability, and mortality rates of ischemic stroke (IS) in the population are increasing, placing a heavy burden on families and society. Although choices for stroke treatment are still limited, the development of drug therapies and mechanical thrombolytic recanalization has led to improvements in IS patients’ recovery. However, due to the limitation of the thrombolytic time window, there is a great demand for developing drugs for acute ischemic stroke.^[2]^

**Figure 1:**
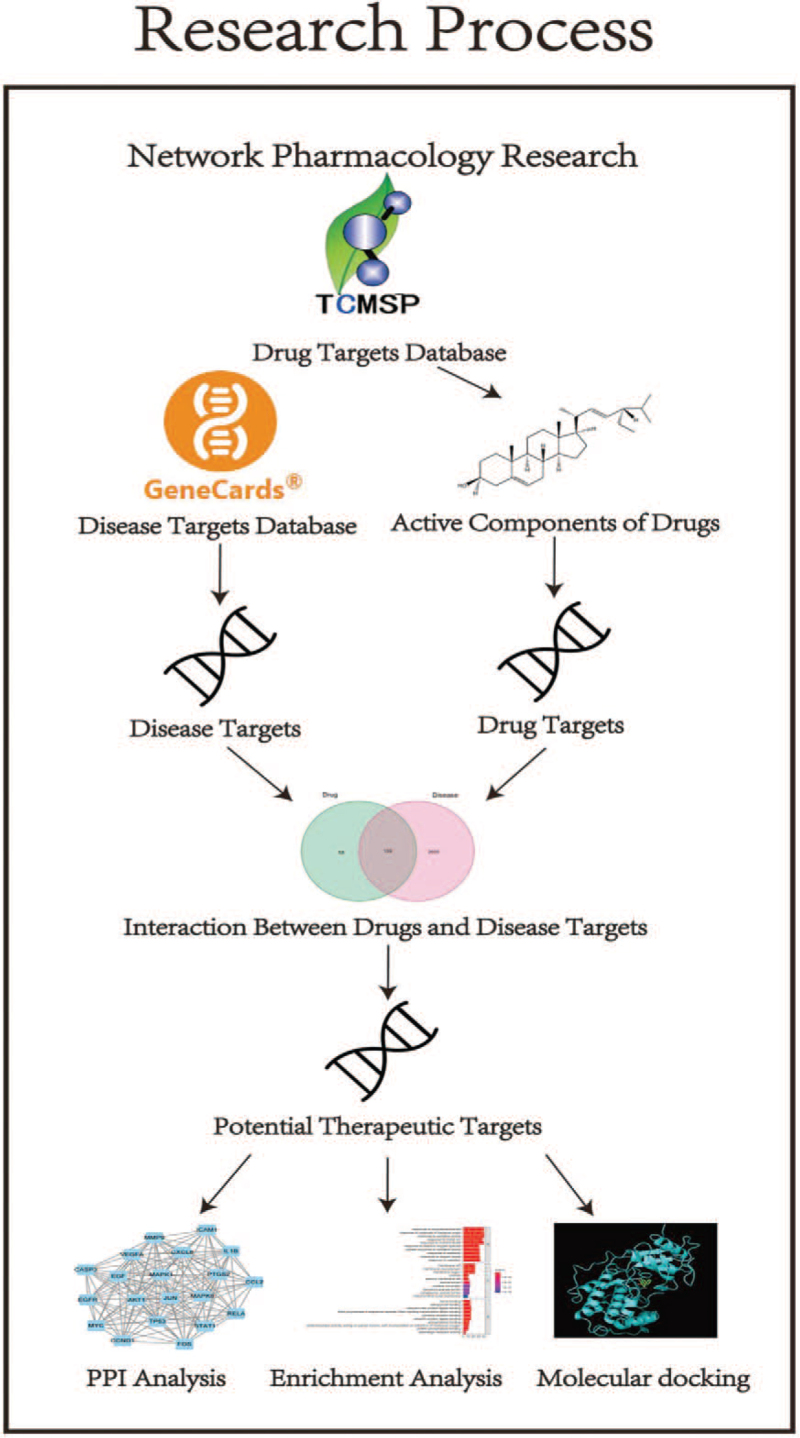
Research process.

After IS occurs, the immunoregulatory response plays a huge role in regulating immune inflammation, oxidative stress, cell damage, apoptosis, and protecting nerve function.^[3]^ Recently, immunomodulatory therapy for IS has become a hot spot in neurology research. The immunomodulatory effects of traditional Chinese medicines on IS have been recently studied in basic and clinical trials.^[4]^ Studies have shown that Qi deficiency and blood stasis syndrome is common type of IS.^[5,6]^ Therefore, strategies that promote blood circulation and remove blood stasis are common methods for treating IS.^[2]^ The proper and reasonable use of drugs that activate blood and remove stasis is important for promoting blood supply to the brain, saving the ischemic penumbra, protecting nerve cells, and regulating the immune response.^[7]^ Basic and clinical research on the efficacy of blood circulation promotion and blood stasis removal for IS treatment has been carried out.^[8]^ Radix Paeoniae Rubra-Flos Carthami herb pair's (RPR-FC) is commonly used for the treatment of IS.^[9]^ It is included in many classical traditional Chinese medicine prescriptions. The combination of the 2 herbs can enhance blood circulation and remove blood stasis.^[9]^ The specific research process is as Figure 1. Traditional Chinese Medicine Systems Pharmacology (TCMSP) database was used to screen the active components of RPR-FC and predict its action targets. The disease targets of ischemic stroke were searched by Genecards and OMIM database, intersected with the action targets of Radix Paeoniae Rubra and the Flos Carthami, screened out the common targets as the research targets, and constructed the protein–protein interaction (PPI) network diagram of the research targets. Enrichment analysis of GO and KEGG pathways. Molecular docking technology was used to verify that RPR-FC has good binding activity with potential targets.

## 2 Materials and methods

### 2.1 Active components and targets

We searched the TCMSP database for the keywords “Radix Paeoniae Rubra” and “Carthami Flos” and used bioavailability (F) ≥30% and drug-likeness (DL) ≥0.18 as criteria.^[10]^

F refers to the percentage of oral doses of a drug that reaches the blood circulatory system, and DL refers to the similarity of the components of a prescription to known drugs. Based on this search, the RPR-FC's effective components and the corresponding targets of the compound were identified from the TCMSP database.

### 2.2 Construction of “Component-Target” network diagram

The UniProt database (http://www.uniprot.org/uniprot/) was used to determine the gene names and UniProt numbers of all the targets and convert them into target abbreviations.^[11]^ The active components of the RPR-FC and their corresponding targets were imported into Cytoscape 3.8 software to construct a “Component-Target” network diagram. In the network diagram, the nodes represent chemical components and targets. The edges represent interactions. The degrees indicate the number of edges connected to a node, and the degree values are positively correlated with function. The “Network Analyzer” was used to analyze and derive the degree values, betweenness, and other topology attributes.

### 2.3 Screening of IS disease targets

Disease targets were identified from the GeneCards database (https://www.genecards.org) and OMIM database (http://omim.org/) using “ischemic stroke” as a keyword.^[12,13]^ Duplicate targets were removed with UniProt. Using the Venny platform (http://bioinfogp.cnb.csic.es/Tools/Venny/), the targets of the active components of the RPR-FC were compared with the IS targets, and the targets of the RPR-FC for the treatment of IS were identified.^[14]^

### 2.4 Construction of the PPI network and core network

The common targets of the RPR-FC and IS were imported into the STRING database (https://string-db.org/).^[15]^ The species was limited to “human,” interactions were determined, and node1, node2, and combined score information were retained in the file. The above data were imported into Cytoscape 3.8 software, a PPI network diagram of the target was constructed, and the core network was screened.^[16]^

### 2.5 Enrichment analysis

R language (ClusterProfiler package) was used to carry out GO enrichment analysis and KEGG enrichment analysis at the biological process, molecular, and cell levels.^[17]^ Graphs and bubble graphs were generated from the data.

### 2.6 Molecular docking study

The 3D structures of the active components of the RPR-FC were downloaded from the TCMSP database, and the 3D structures of the candidate IS targets were downloaded from the PDB database. The targets’ structure was modified by removing ligands, water, and hydrogen by importing them into PyMOL 2.4 software. Using AutoDock 4.0 software, the candidate pharmacodynamic components and candidate targets were used for docking studies after preprocessing.^[18]^ In this study, the active components of the RPR-FC to effectively bind potential therapeutic targets were verified by molecular docking studies, and the docking results were analyzed to verify the reliability of this research.

## 3 Results

### 3.1 Screening of main active components and targets

Using the TCMSP database for screening, 29 active components of Paeoniae Rubra, and 15 active components of Flos Carthami were identified (Table 1). A total of 498 effective targets, 159 of which were targets of Radix Paeoniae Rubra and 339 of which were targets of Flos Carthami, were identified by analyzing the herb components’ corresponding targets. The abovementioned effective components and targets were compared and combined. After UniProt correction and comprehensive collation, 197 effective targets of the RPR-FC were identified.

**Table 1:** Active components of the Radix Paeoniae Rubra-Flos Carthami herb pair.

Herbs	molId	molName	F	DL
Radix Paeoniae Rubra	MOL007004	albiflorin	30.25	0.77
	MOL006990	(1S,2S,4R)-trans-2-hydroxy-1,8-cineole-B-d-glucopyranoside	30.25	0.27
	MOL007003	benzoyl paeoniflorin	31.14	0.54
	MOL007025	isobenzoylpaeoniflorin	31.14	0.54
	MOL007014	8-debenzoylpaeonidanin	31.74	0.45
	MOL002883	ethyl oleate (NF)	32.4	0.19
	MOL002714	baicalein	33.52	0.21
	MOL006994	1-o-beta-d-glucopyranosyl-8-o-benzoylpaeonisuffrone_qtβ	36.01	0.3
	MOL000359	sitosterol	36.91	0.75
	MOL000358	beta-sitosterol	36.91	0.75
	MOL006999	stigmast-7-en-3-ol	37.42	0.75
	MOL005043	campest-5-en-3beta-ol	37.58	0.71
	MOL002776	baicalin	40.12	0.75
	MOL004355	spinasterol	42.98	0.76
	MOL001002	ellagic acid	43.06	0.43
	MOL000449	stigmasterol	43.83	0.76
	MOL007005	albiflorin_qt	48.7	0.33
	MOL001921	lactiflorin	49.12	0.8
	MOL001924	paeoniflorin	53.87	0.79
	MOL000492	(+)-catechin	54.83	0.24
	MOL007012	4-o-methyl-paeoniflorin_qt	56.7	0.43
	MOL007008	4-ethyl-paeoniflorin_qt	56.87	0.44
	MOL006992	(2R,3R)-4-methoxyl-distylin	59.98	0.3
	MOL007018	9-ethyl-neo-paeoniaflorin A_qt	64.42	0.3
	MOL007022	evofolin	64.74	0.22
	MOL006996	1-o-beta-d-glucopyranosylpaeonisuffrone_qt	65.08	0.35
	MOL007016	paeoniflorigenone	65.33	0.37
	MOL001925	paeoniflorin_qt	68.18	0.4
	MOL001918	paeoniflorgenone	87.59	0.37
Carthami Flos	MOL002757	7,8-dimethyl-1H-pyrimido[5,6-g]quinoxaline-2,4-dione	45.75	0.19
	MOL002717	qt_carthamone	51.03	0.2
	MOL002714	baicalein	33.52	0.21
	MOL000422	kaempferol	41.88	0.24
	MOL002719	6-hydroxynaringenin	33.23	0.24
	MOL000006	6-hydroxynaringenin	36.16	0.25
	MOL002712	6-hydroxykaempferol	62.13	0.27
	MOL000098	quercetin	46.43	0.28
	MOL002721	quercetagetin	45.01	0.31
	MOL002698	lupeol-palmitate	33.98	0.32
	MOL002710	pyrethrin II	48.36	0.35
	MOL002710	[(E)-4-(3,5-dimethoxy-4-oxo-1-cyclohexa-2,5-dienylidene)but-2-enylidene]-2,6-dimethoxycyclohexa-2,5-dien-1-one	48.47	0.36
	MOL002707	[(E)-4-(3,5-dimethoxy-4-oxo-1-cyclohexa-2,5-dienylidene)but-2-enylidene]-2,6-dimethoxycyclohexa-2,5-dien-1-one	43.18	0.5
	MOL002707	phytoene	39.56	0.5
	MOL002680	flavoxanthin	60.41	0.56

### 3.2 “Component-Target” network analysis

**Figure 2:**
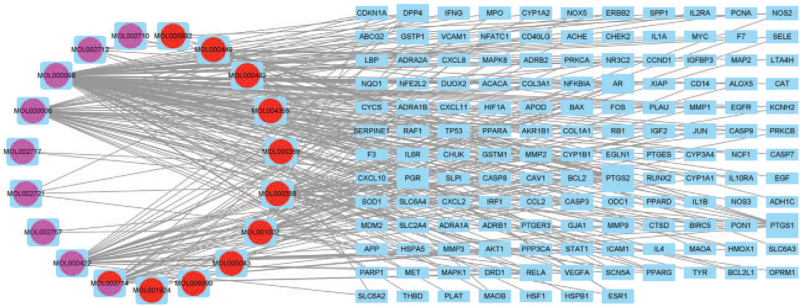
“Component-Target” network diagram.

The candidate components and the corresponding targets of the RPR-FC were imported into Cytoscape 3.8 software to construct a “Component-Target” network diagram. The scattered nodes were removed, and a total of 158 nodes and 283 edges remained. The circle on the left side of the figure represents the active components of RPR-FC. The red circles indicate the active components of Radix Paeoniae Rubra, and the purple circles indicate the active components of Flos Carthami. The circle containing 2 colors indicates the common active components of the RPR-FC. The blue nodes on the right represent drug targets. The larger the node is, the more components are connected to the gene. Each edge represents an interaction between the component and the target (Fig. 2).

### 3.3 Prediction of therapeutic targets and construction of the PPI core network

**Figure 3:**
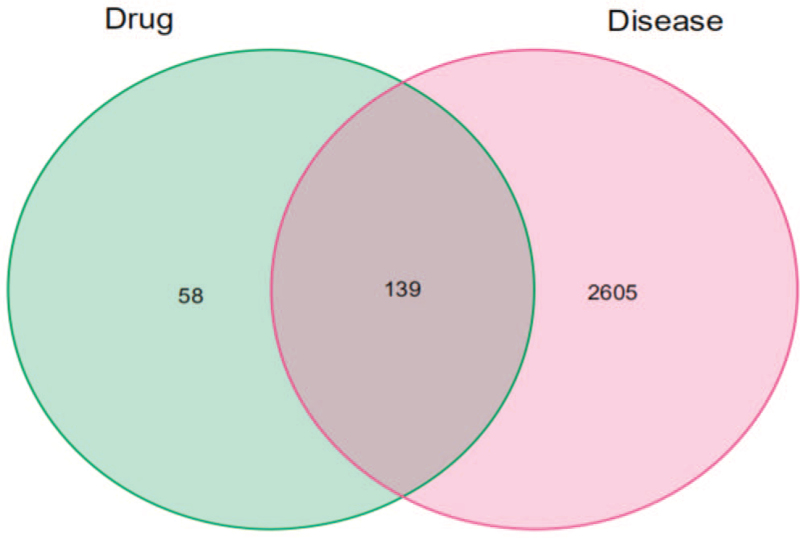
“Component-Target” network diagram. The green circle represents drug targets, the pink circles represent disease targets, and the overlapping area represents potential therapeutic targets.

**Figure 4:**
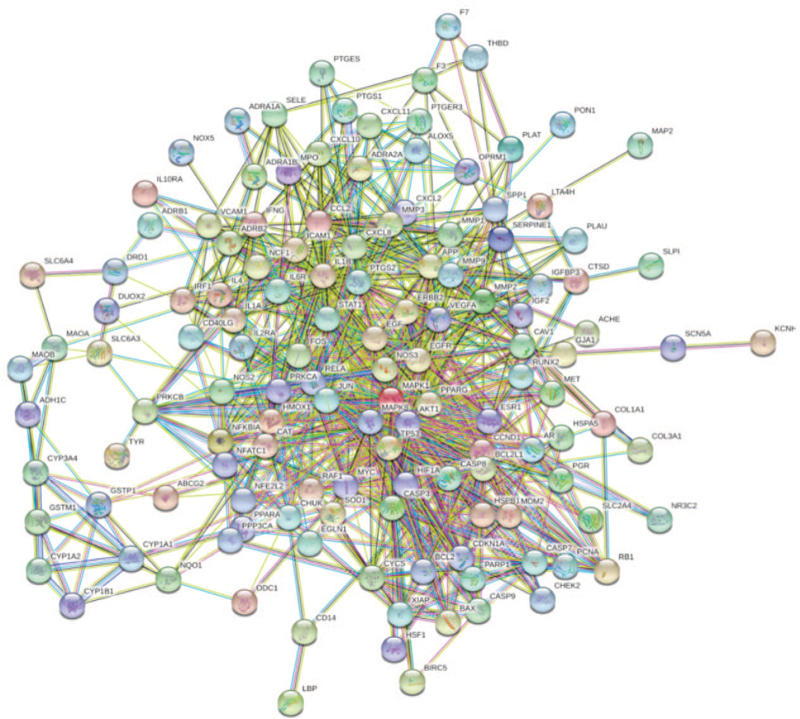
Construction of a PPI network diagram. The circles are labeled with the targets’ abbreviations, and the straight lines represent associations between the targets. The darker the color, the higher the correlation. PPI = protein–protein interaction.

**Figure 5:**
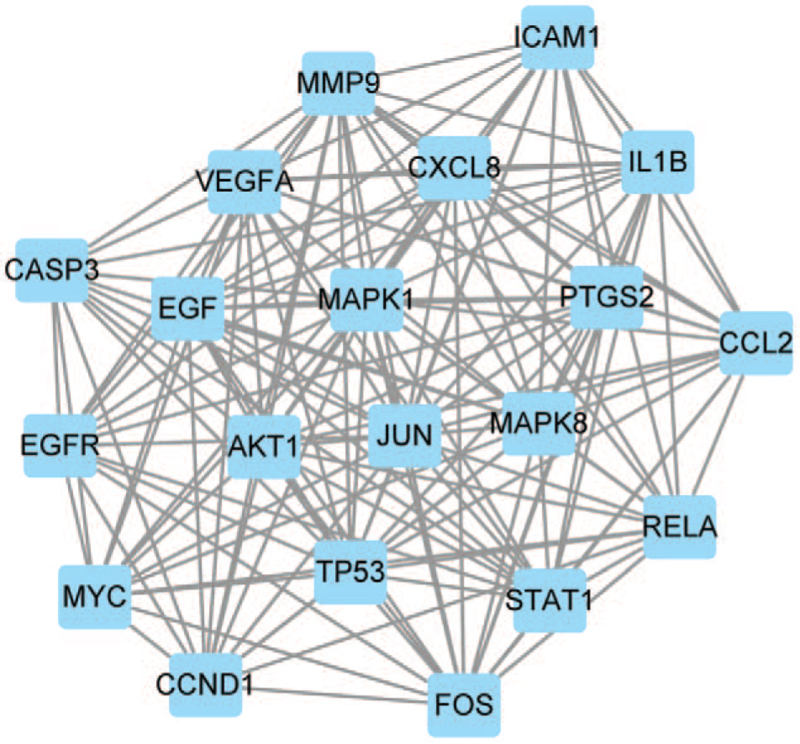
Screening of the PPI core network. The blue squares are the selected core targets. PPI = protein–protein interaction.

Using “Ischemic stroke” as the keyword, a total of 2744 IS-related targets were identified from the GeneCards database and OMIM database. The 197 drug targets and the 2744 disease targets were mapped with Venny 2.1.0, and 139 common “Drug-Disease” targets were obtained (Fig. 3). The 139 common targets were imported into the STRING platform for PPI network analysis and Cytoscape 3.8 for visualization processing. The resulting PPI network had 150 edges (Fig. 4). The above targets were screened based on betweenness, closeness, eigenvector, local average connectivity, network and degree, and 20 nodes with the highest parameters considered the network's key genes were identified. These nodes were JUN, MAPK1, TP53, MAPK8, AKT1, VEGFA, MMP9, EGF, CXCL8, FOS, PTGS2, IL1B, ICAM1, STAT1, RELA, CCL2, CCND1, MYC, EGFR, and CASP3 (Fig. 5).

### 3.4 GO enrichment and KEGG analysis

**Figure 6:**
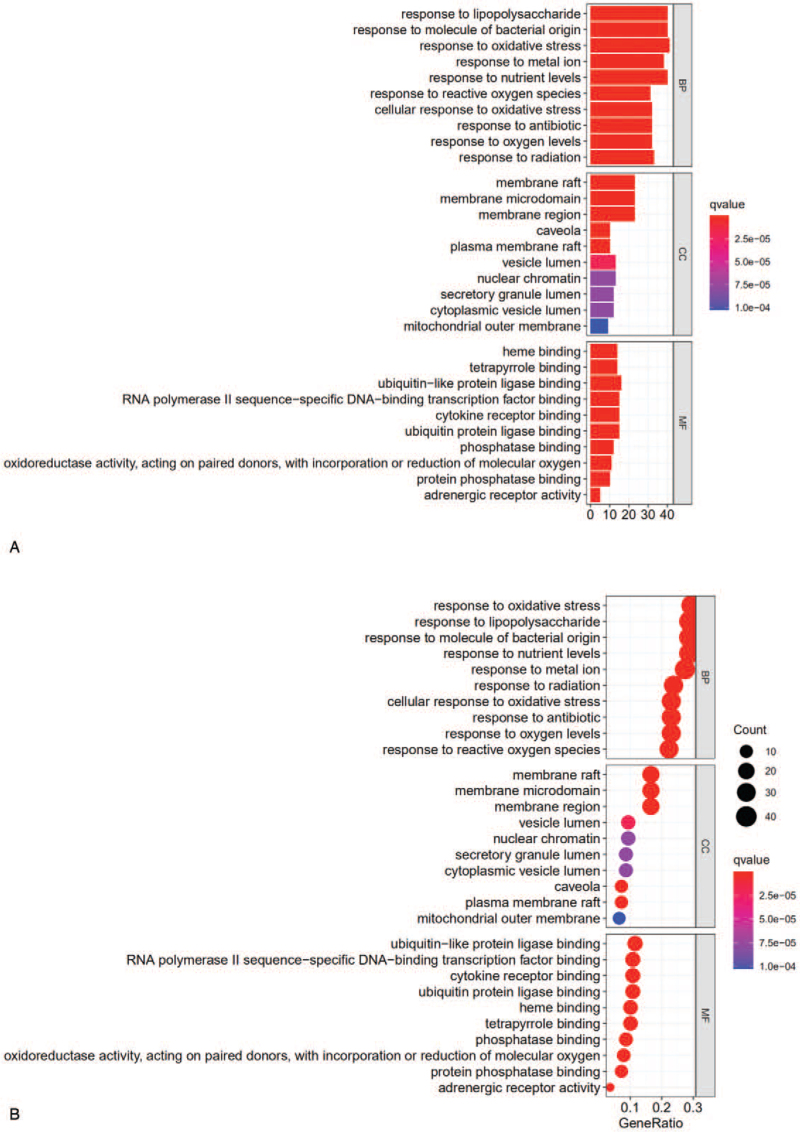
Enrichment analysis of potential therapeutic targets. (A) Bar chart of the GO enrichment analysis results. (B) Dot chart of the GO enrichment analysis results. (C) Bar chart of the KEGG pathway analysis results. (D) Dot chart of the KEGG pathway analysis results. In the bar chart, the abscissa represents the number of targets, and the ordinate shows the names of the enriched terms. The redder the color is, the smaller the adjusted *P* value is. The bluer the color is, the larger the adjusted *P* value is. In the dot chart, the abscissa represents the ratio of targets, and the ordinate shows the names of the enriched terms. The larger the circle is, the greater the enrichment is, and vice versa. The redder the circle is, the smaller the adjusted *P* value is. The bluer the circle is, the larger the adjusted *P* value is.

R language (cluster profiler package) was used to analyze the enrichment of GO terms and KEGG pathways (*P* < .05). There were 2252 biological processentries, 183 molecular functions entries, and 72 cellular components entries identified by GO enrichment analysis. GO analysis revealed that the above targets play a biological role through the lipopolysaccharide reaction, oxidative stress response, etc. The proportion of cellular components terms in the intercellular space was large. Ubiquitin-like protein ligase binding (ubiquitin) was the most enriched molecular functions terms. The top 10 terms, according to corrected *P* values are displayed in the graphic in Figure 6 (A and B).

A total of 160 KEGG pathways (*P* < .05), including fluid shear stress and atherosclerosis, the AGE-RAGE signaling pathway in diabetic compositions, the TNF signaling pathway, and the IL-17 signaling pathway were identified. The top 30 pathways are shown in the graphic in Figure 6 (C and D). We screened pathways and found that the RPR-FC may be involved in fluid shear stress and the atherosclerosis signaling pathway, the MAPK signaling pathway, the NF-kappa B signaling pathway, the phosphoinositide 3-kinase (PI3K)-Akt signaling pathway, the VEGF signaling pathway, the TNF signaling pathway, and other signaling pathways to exert immunoregulatory and neuroprotective effects after IS. A literature search on the above pathways showed that acute cerebral ischemic events and thrombosis are related to carotid atherosclerotic plaque rupture/erosion. Higher wall shear stress values are related to the presence of calcification. Fluid shear stress and atherosclerosis regulate the wall and play an important role in high stress.^[19]^ The TNF signaling pathway mainly regulates the inflammatory response after cerebral infarction. Clinical studies have shown that inflammatory cytokines are effective in the pharmacological regulation of cerebral infarction. Experimental evidence has shown that tumor necrosis is the main target for new stroke treatments.^[20]^ The MAPK signaling pathway regulates gene expression and basic cellular processes, such as cell proliferation, differentiation, migration, metabolism, and apoptosis, in eukaryotic cells. It is considered a therapeutic target for many diseases. To date, increasing evidence has shown that the MAPK signaling pathway is involved in the pathogenesis and development of IS.^[21]^ IL-17 regulates stem cells and the generation of cortical neural stem cells (NSCs) in adults after stroke. Knockout of IL-17 helps to regulate the PI3K-Akt pathway, promotes the proliferation of NSCs, and promotes nerve growth after IS.^[22]^ This study found that the RPR-FC has neuroprotective effects, promoting nerve repair, vascular remodeling, inhibition of neuronal apoptosis, and regulation of inflammatory factor expression after IS.

### 3.5 Consolidation and construction of the “Component-Target-Pathway” network

KEGG enrichment analysis was performed to construct a signaling pathway map, and the related literature was reviewed. The 5 signaling pathways most related to IS are displayed in the graphic in Figure 7   (A-E). Six potential components and 5 signaling pathways corresponding to the 20 key targets of the RPR-FC were combined into a “Component-Target-Pathway” network diagram, as shown in Figure 8. This diagram reflects the multi-component, multi-target, and multi-pathway characteristics of the RPR-FC in the comprehensive treatment of IS.

**Figure 7:**
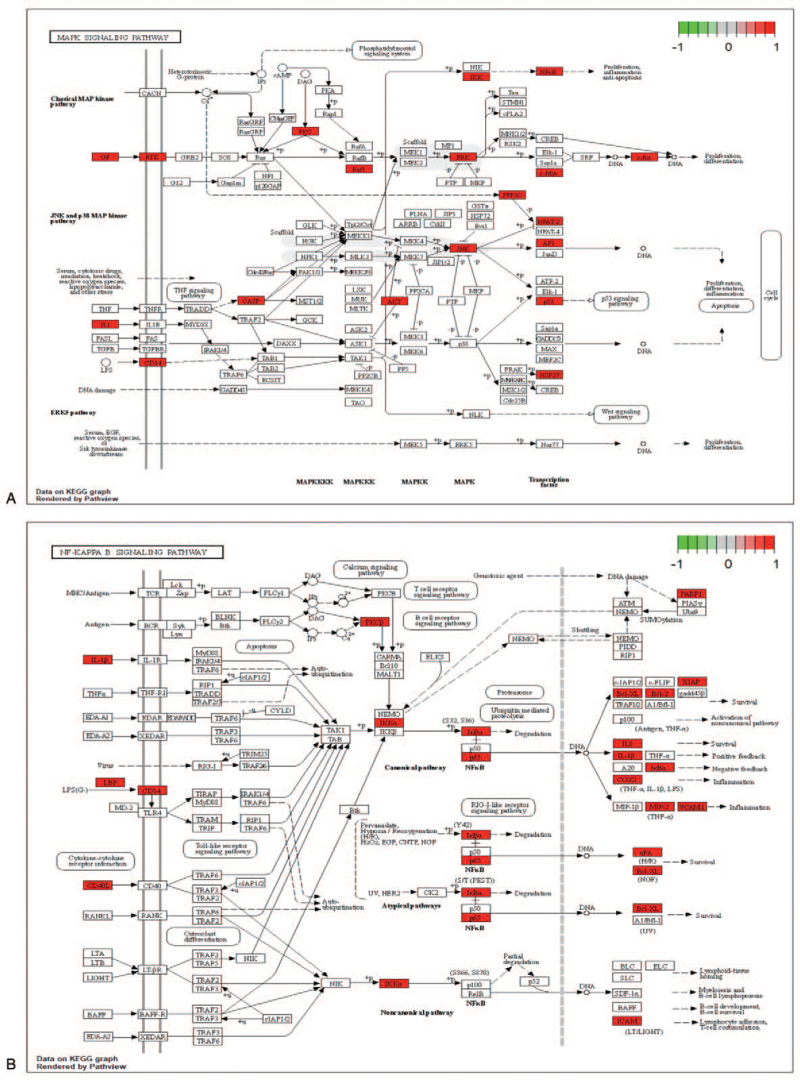
KEGG pathway diagram. (A) MAPK signaling pathway. (B) NF-kappa B signaling pathway. (C) PI3K-Akt signaling pathway. (D) VEGF signaling pathway. (E) TNF signaling pathway. The red nodes indicate relevant enriched targets.

**Figure 7 (Continued):**
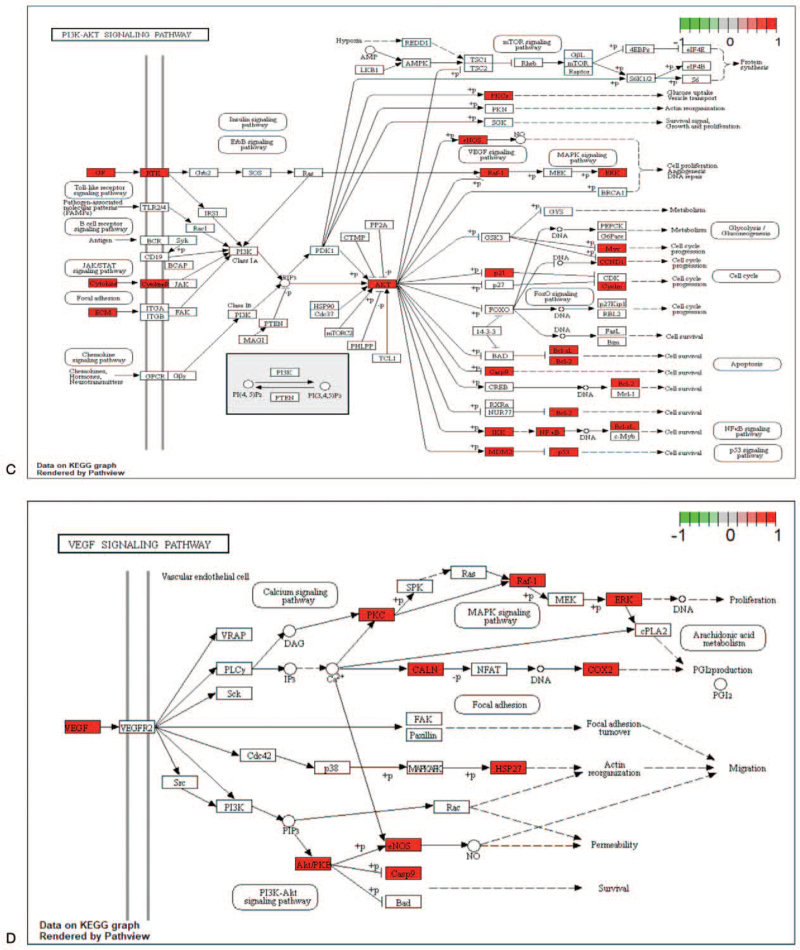
KEGG pathway diagram. (A) MAPK signaling pathway. (B) NF-kappa B signaling pathway. (C) PI3K-Akt signaling pathway. (D) VEGF signaling pathway. (E) TNF signaling pathway. The red nodes indicate relevant enriched targets.

**Figure 7 (Continued):**
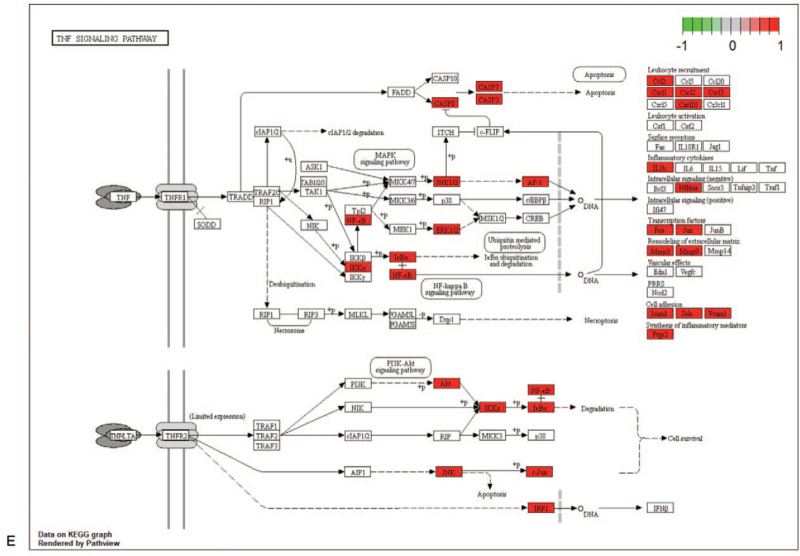
KEGG pathway diagram. (A) MAPK signaling pathway. (B) NF-kappa B signaling pathway. (C) PI3K-Akt signaling pathway. (D) VEGF signaling pathway. (E) TNF signaling pathway. The red nodes indicate relevant enriched targets.

**Figure 8:**
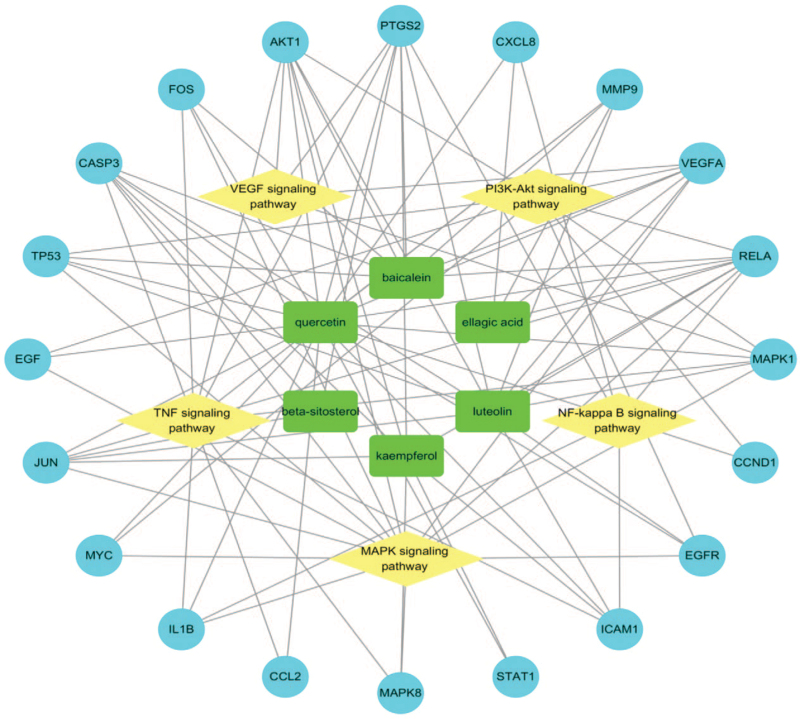
“Component-Target-Pathway” diagram. The green squares are the active component of the Radix Paeoniae Rubra-Flos Carthami herb pair, the blue circles are the targets, and the yellow diamonds are the enriched pathways.

### 3.6 Molecular docking

The PDB database (http://www.rcsb.org) was used to download the 3-dimensional structures of the proteins, PyMOL 2.4 software was used to optimize the protein receptors, and AutoDock 4.0 was used for component docking. In the “Component-Target-Pathway” network diagram, the 4 components with the highest degrees, namely, luteolin, quercetin, kaempferol, and baicalein, were selected as candidate therapeutic docking components. The targets with the highest degrees, namely, MAPK1, AKT1, VEGFA, and CASP3, were selected as candidate docking targets. Molecular docking analysis of these targets was performed, and the docking scores were calculated. A scoreless than the original ligand score indicated that the component has good docking activity with the target and verified the docking result of the chemical component. The relevant information of the molecular docking model is shown in Table 2. The relevant results of the target and the original ligand docking are shown in Figure 9  (A-D). The results show that the components of the RPR-FC have good docking activity with potential therapeutic targets.

**Table 2:** Docking score.

Name of compound	Target name	Number of original gametes
Luteolin	MAPK1	−8.4
Quercetin	AKT1	−8.8
Kaempferol	VEGFA	−8.5
Baicalein	CASP3	−7.5

**Figure 9:**
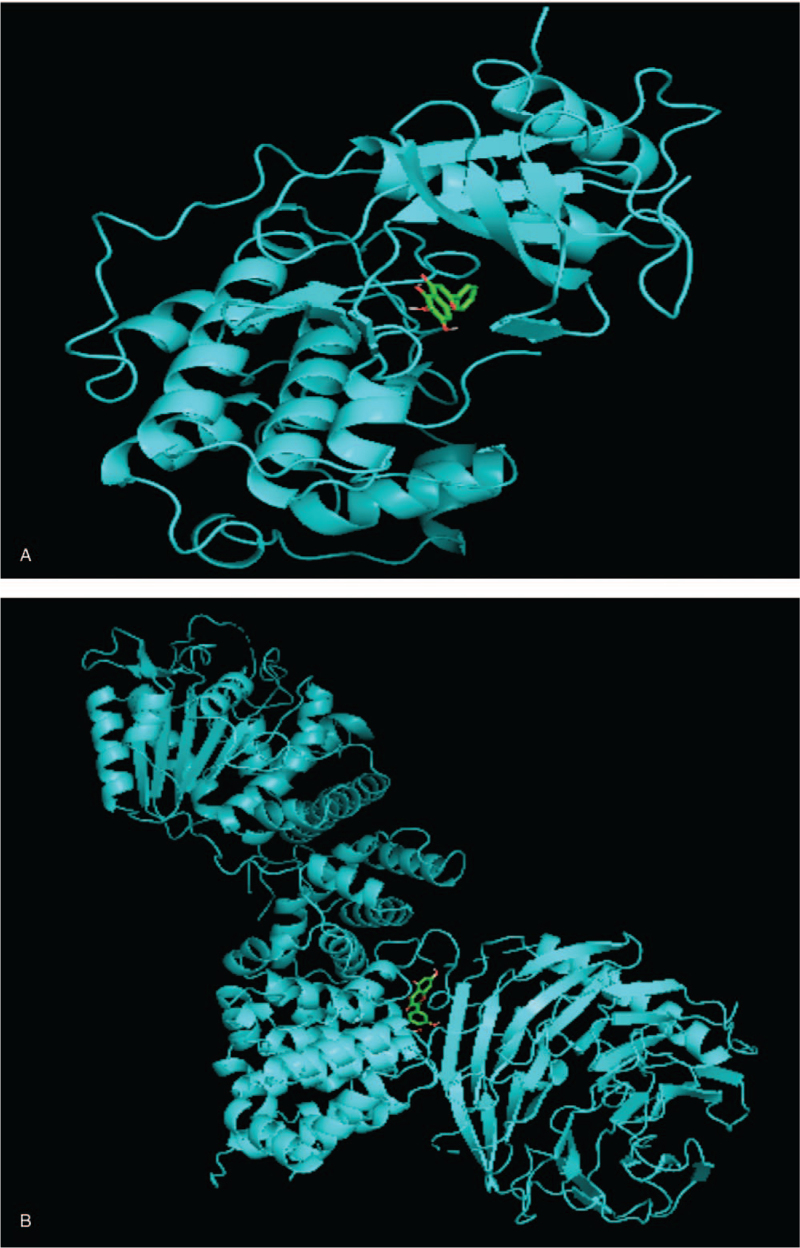
Docking of the target and the original ligand. (A)Luteolin-MAPK1. (B) Quercetin-AKT1. (C) Kaempferol-VEGF. (D) Baicalein-CASP3.

**Figure 9 (Continued):**
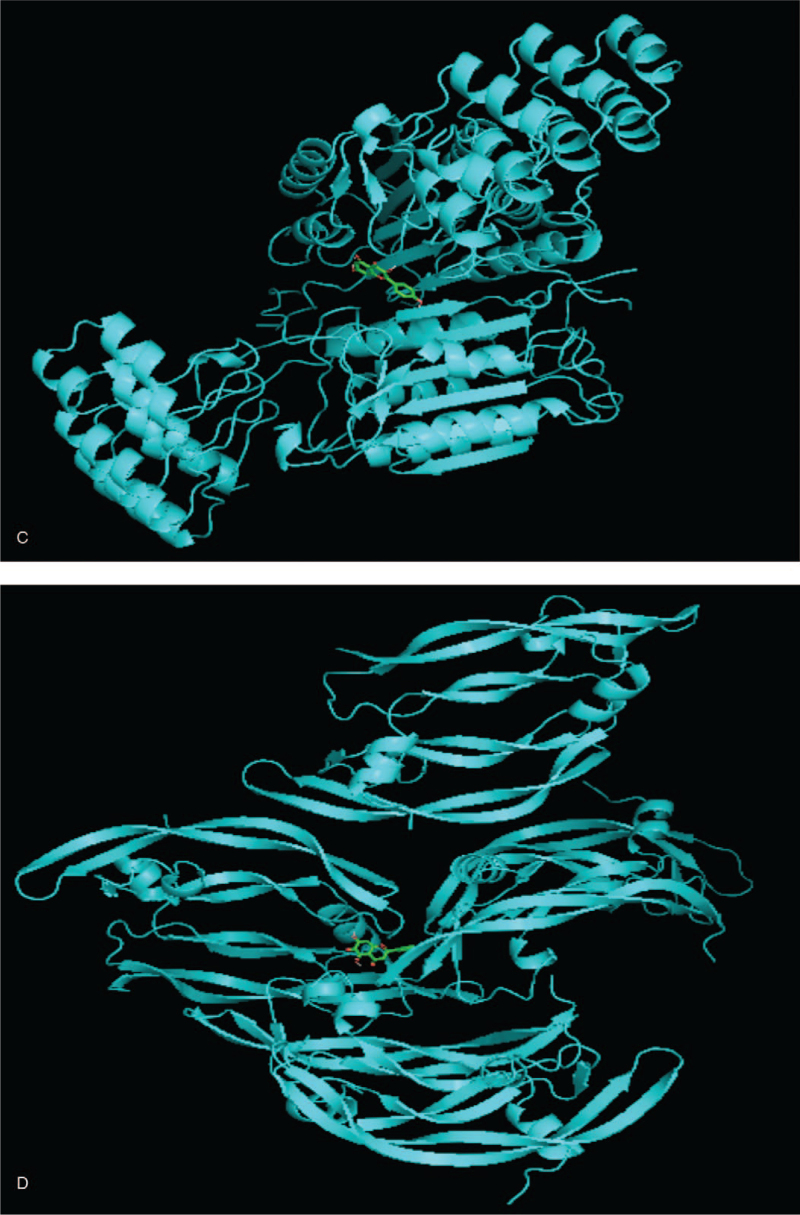
Docking of the target and the original ligand. (A)Luteolin-MAPK1. (B) Quercetin-AKT1. (C) Kaempferol-VEGF. (D) Baicalein-CASP3.

## 4 Discussion

Modern pharmacological studies have shown that the effects of some active components of Chinese herbal medicines against IS are related to anti-inflammatory, anti-oxidative stress, and nerve cell protection functions. For example, Chuanxiong can regulate IS-related targets, biological processes, and signaling pathways. Animal experiments have shown that Chuanxiong can improve the neurobehavioural scores of IS rats and has a protective effect on neurons (*P* < .05).^[23]^*Salvia miltiorrhiza* can alleviate IS by promoting the survival of mesenchymal stem cells. In rats subjected to middle cerebral artery occlusion, treatment with *S. miltiorrhiza* can lead to recovery of the infarct area and positive changes in rat behavior.^[24]^ The RPR-FC is a common combination used for the treatment of cerebrovascular diseases and is a component in traditional Chinese medicine prescriptions such as Xuefu Zhuyu Decoction and Buyang Huanwu Decoction.^[25]^ Radix Paeoniae Rubra is sour and soft in nature.^[26]^ It has the functions of nourishing yin, promoting blood stasis, relieving pain, and cooling blood. Its main chemical components include paeoniflorin, paeoniflorin, paeonol, and *S. miltiorrhiza*. These components regulate NF-kappa B and other signaling pathways.^[27]^ For example, Paeoniae Radix Rubra can significantly reduce the volume of the cerebral infarct caused by ischemic injury and alleviate neuronal damage caused by transient cerebral ischemia by inhibiting glial hyperplasia and improving antioxidant activity.^[28]^ Radix Paeoniae Rubra is pungent, warm, and non-toxic and activates blood circulation, removed blood stasis, and relieves pain. Its main components are safflower yellow pigment, quercetin, kaempferol, and other components, which can regulate the NF-kappa B signaling pathway, the TNF signaling pathway, and other signal pathways to exert anti-inflammatory effects and prevent and treat cardiovascular and cerebrovascular diseases.^[29]^ For example, hydroxysafflor yellow A confers neuroprotection against focal cerebral ischemia by modulating the crosstalk between the JAK2/STAT3 and SOCS3 signaling pathways.^[30]^ The RPR-FC is a commonly used drug pair for the treatment of IS. The 2 herbs are able to enhance blood circulation and remove blood stasis. Both Radix Paeoniae Rubra and Flos Carthami extract have anti-inflammatory and neuroprotective effects against IS, but there is no literature on the mechanism of these 2 traditional Chinese medicines in the treatment of IS.^[31]^ Therefore, this study used network pharmacology combined with molecular docking analysis to study the mechanism of the RPR-FC in the treatment of IS to provide a scientific basis for the use of treatment strategies that promote blood circulation and remove blood stasis for immunoregulation in IS.

In this study, 10 key targets with high degree values, that is, JUN, MAPK1, TP53, MAPK8, AKT1, VEGFA, MMP9, EGF, CXCL8, FOS, PTGS2, IL1B, STAT1, RELA, CCL2, CCND1, MYC, EGFR, and CASP3, were identified after screening the PPI network. It can be speculated that the RPR-FC's effective components may exert their effects through these targets. JUN is the transcription factor of AP-1, which can lead to steroid production and increase gene expression after the CAMP signaling pathway is stimulated. Studies have shown that CAMP is closely related to axonal regeneration after stroke.^[32]^ As a member of the gene-encoding MAP kinase family, MAPK1 integrates a variety of biochemical signals and participates in a variety of cellular processes, such as proliferation, differentiation, transcriptional regulation, and development.^[33]^ The protein encoded by the TP53 gene responds to a variety of cellular stresses to regulate the expression of target genes, thereby inducing cell cycle arrest, apoptosis, senescence, DNA repair, or metabolic changes. Studies have shown that the methylation level of the TP53 promoter is related to neck arterial intima-media thickness and that the degree of carotid atherosclerosis and the circulating level of homocysteine in the peripheral blood are related, which indicates that TP53 is related to the pathophysiology of IS.^[34]^ AKT1 is a serine-threonine protein kinase. After being phosphorylated by PI3K, AKT/PI3K acts as a key component of many signaling pathways and is regulated by signaling pathways, such as the HIF-α signaling pathway and the TNF signaling pathway. VEGFA is vascular endothelial growth factor A. VEGFA can promote the proliferation and division of vascular endothelial cells after cerebral ischemia. VEGFA signaling clearly regulates angiogenesis after IS and reduces the degree of cerebral ischemia.^[35]^ CASP3 and signal transducer and activator of transcription 3 target VEGFA to regulate angiogenesis after cerebral ischemia and can also control the survival and regeneration of nerve cells.^[36]^ TNF-α is a proinflammatory cytokine that can aggravate the inflammatory response of neutrophils. PTGS2 is a prostaglandin peroxidase that can be induced by inflammatory mediators, cytokines, and other in vivo and in vitro factors. Specific downregulation of PTGS2 expression can inhibit the NF-kappa B signaling pathway, thus inhibiting the apoptosis of EPCs and promoting the proliferation, migration, and angiogenesis of EPCs, and has a protective effect against IS in mice.^[37]^

KEGG pathway analysis and a large number of studies have indicated that the MAPK signaling pathway, NF-kappa B signaling pathway, PI3K-Akt signaling pathway, and VEGF signaling pathway may be the 4 key signaling pathways through which the RPR-FC can treat IS. Among these pathways, the MAPK signaling pathway responds to a variety of stimuli and transmits signals from the cell membrane to the nucleus. This pathway controls a wide range of cellular processes, including growth, inflammation, and stress response. Increasing evidence has shown that MAPK is an important regulator of ischemic and hemorrhagic cerebrovascular diseases, increasing its potential as a drug target for stroke.^[38]^ The NF-kappa B protein family is a pleiotropic transcription factor that can specifically bind to the KB site of a variety of promoters, thereby promoting their transcription and expression. It is affected by oxidative stress, bacterial lipopolysaccharide, and cytokines. After activation of various stimuli, NF-kappa B can regulate the production of inflammatory cytokines, cell surface receptors, transcription factors, adhesion molecules, etc. The NF-kappa B pathway can affect the cell cycle of brain cells by regulating the apoptosis rate of cells and plays a regulatory role in IS.^[39]^ The tyrosine kinase PI3K-Akt signaling pathway may be an important signal transduction pathway for the survival of cerebral ischemic neurons.^[40]^ It is regulated by most neurotransmitters in cerebellar neurons, sympathetic neurons, sensory neurons, and cortical motor neurons. PI3K plays a role in cell survival plays. Cell experiments have shown that luteolin can treat IS by improving cell viability and reducing cell apoptosis by activating the PI3K/Akt signaling pathway.^[41]^ The VEGF family is known to regulate angiogenesis. In the brain, VEGF is an important regulator of angiogenesis, neuroprotection, and neurogenesis. VEGF can directly contribute to the proliferation and angiogenesis of vascular endothelial cells in ischemic and hypoxic tissues or organs.^[42]^ Sun found that when the level of VEGF increases, the numbers of NSCs, neurons, and astrocytes increase but that when the level of VEGF decreases, and the numbers of these 3 types of cells also decrease.^[43]^ The effect of promoting the proliferation and differentiation of endogenous NSCs after cerebral infarction has become a popular topic of research, and VEGF has been shown to play an important role in the proliferation and differentiation of NSCs.^[44]^

The active components of the RPR-FC that bind best to the key targets are quercetin, luteolin, kaempferol, and baicalein. Quercetin is a flavonoid compound extracted from food that plays an anti-atherosclerotic role, by exerting anti-inflammatory, antioxidant, and endothelial-dependent vasodilatory effects and lowering blood lipids.^[45]^ Studies have shown that quercetin can regulate the AMPK signaling pathway, PI3K/Akt/IKKα/NF-kappaB signaling pathway, and other signaling pathways, which are closely related to IS.^[46,47]^ Luteolin can inhibit neuronal cell degeneration and alleviate motor and sensory dysfunction. In vitro experiments have shown that luteolin can alleviate the decline in neuronal cell viability caused by microglial activation, protect against endothelial cell damage induced by oxidative stress, and protect against atherosclerotic diseases.^[48,49]^ Kaempferol is the most important flavonoid in *Carthamus tinctorius*. Studies on NF-kappa B, TNF-α, IL-6, oxidative stress, and cardiovascular injury have confirmed that kaempferol protects the vascular endothelium and have revealed that its specific mechanism may be related to the Nrf2/HO-1 signaling pathway.^[50]^ Kaempferol can reduce the injury volume after ischemia–reperfusion (I/R) injury for 90 minutes and has a protective effect on mitochondria in the area of brain injury.^[51]^ Recent studies have shown that kaempferol may prevent ischemic brain injury and neuroinflammation by inhibiting the activation of STAT3 and NF-kappa B, thereby exerting a protective effect against ischemic brain injury.^[52]^ Baicalein is a widely distributed natural flavonoid that has many beneficial pharmacological effects, such as anti-inflammatory and antioxidant effects. Animal experiments have shown that baicalein can significantly reduce Nrf2 and AMPK levels, suggesting that baicalein may play a neuroprotective role by downregulating the expression of NF-kappa B and LOX-1 and inhibiting the AMPK/Nrf2 pathway.^[53]^

## 5 Conclusions

At present, the RPR-FC is widely used for the treatment of IS, but pharmacological research reports are scarce. The active components of the drug pair such as quercetin, luteolin, kaempferol, and baicalein may act on MAPK1, TP53, TP53, TP53, and MAPK through IS-related pathways, including the MAPK signaling pathway, NF-kappa B signaling pathway, PI3K-Akt signaling pathway, and VEGF signaling pathway. MAPK8, AKT1, VEGFA, MMP9, EGF, and other targets exert anti-inflammatory, immunomodulatory, anti-thrombotic, and vascular endothelium protective effects to treat IS. In view of the limitations of network pharmacology, it is necessary to further experimentally verify the potential active components, targets, and related pathways of the RPR-FC in the future.

## Author contributions

**Conceptualization:** Dexi Zhao

**Formal analysis:** Xingyu Chen

**Funding acquisition:** Dexi Zhao, Yue Wang

**Project administration:** Ruonan Wang

**Visualization:** Xingyu Chen, Ying Ma

**Writing – original draft:** Xingyu Chen

**Writing – review & editing:** Xingyu Chen, Ying Ma
